# Gaia: An AI-enabled genomic context–aware platform for protein sequence annotation

**DOI:** 10.1126/sciadv.adv5109

**Published:** 2025-06-20

**Authors:** Nishant Jha, Joshua Kravitz, Jacob West-Roberts, Cong Lu, Antonio Pedro Camargo, Simon Roux, Andre Cornman, Yunha Hwang

**Affiliations:** ^1^ Tatta Bio, Cambridge, MA 02142, USA.; ^2^ DOE Joint Genome Institute, Lawrence Berkeley National Laboratory, Berkeley, CA 94720, USA.

## Abstract

Protein sequence similarity search is fundamental to biology research, but current methods are typically not able to consider crucial genomic context information indicative of protein function, especially in microbial systems. Here, we present Gaia (Genomic AI Annotator), a sequence annotation platform that enables rapid, context-aware protein sequence search across genomic datasets. Gaia leverages gLM2, a mixed-modality genomic language model trained on both amino acid sequences and their genomic neighborhoods to generate embeddings that integrate sequence-structure-context information. This approach allows for the identification of functionally and/or evolutionarily related genes that are found in conserved genomic contexts, which may be missed by traditional sequence- or structure-based search alone. Gaia enables real-time search of a curated database comprising more than 85 million protein clusters from 131,744 microbial genomes. We compare the homolog retrieval performance of Gaia search against other embedding and alignment-based approaches. We provide Gaia as a web-based, freely available tool.

## Teaser

The genomic language model enables rapid genomic context–aware search and discovery of microbial proteins.

## INTRODUCTION

Protein sequence similarity search is a foundational analytical technique in genomics research, critical for inferring protein function and finding evolutionary relationships. Tools like BLAST (*1*) and HMMER (*2*) have been widely used for these analyses, relying on sequence alignment methods to compare individual query sequences against databases of known proteins. While highly effective for detecting close homologs, these approaches can struggle to identify remote homologs that have diverged across large evolutionary distances.

Structural search methods are an effective alternative method to detect remote homology between proteins. Tools like Foldseek (*3*) perform structural similarity search on large databases of experimental and in silico–predicted protein structures including the Protein Data Bank (PDB) (*4*) and AlphaFold Database (AlphaFoldDB) (*5*) and are capable of identifying highly similar structures with low sequence similarity to the provided input. However, relying on structural similarities results in limited insights for some intrinsically disordered proteins (*6*, *7*), proteins with conformational changes (*8*), and proteins with low-confidence predicted structures (*9*). In addition, structural search (*6*, *7*) requires precomputed protein structures across large-scale databases, incurring massive computational costs as protein databases grow to the billion scale.

In recent years, the advent of biological language models has opened new avenues for sequence analysis. Models like ESM2 (*10*) and ESM3 (*11*) have shown promise in capturing complex patterns in protein sequences, enabling more sensitive remote homology detection. ESMFold, for example, provides accurate and scalable protein structure prediction using only individual sequences as input. Tools like ProtTrek (*12*), pLM-BLAST (*13*), and PLMSearch (*14*) use protein language models for remote homology detection (*15*) by comparing against databases like Swiss-Prot (*16*) and ECOD (*17*). Embedding-based search is highly efficient and scalable, showing similar or superior performance to alignment-based tools with increased sensitivity (*14*). These approaches complement existing methods, offering alternative perspectives on protein relationships and functions.

However, a critical aspect often overlooked in current search methodologies is the genomic context of proteins, particularly informative of function in microbial genomics. Understanding the genomic neighborhood of a gene can provide crucial insights into its function and regulation. For example, the identification of CRISPR-associated genes relies on analyzing surrounding genomic regions (*18*). Similarly, defense islands are identified by recognizing clusters of genes with shared annotations; “islands” are defined as such by the colocation of these defense genes on the host genome (*19*, *20*). Traditionally, studies and analyses that use genome context are limited in throughput because of the slow, often manual, nature of the work required to inspect a full genomic locus and all the annotations within it.

To address these challenges and bridge the gap between sequence, structure, and context-based analyses, we developed Gaia (Genomic AI Annotator). Gaia is an embedding-based search platform that enables fast and context-aware protein sequence similarity search over large genomic datasets.

## RESULTS

**Fig. 1.**
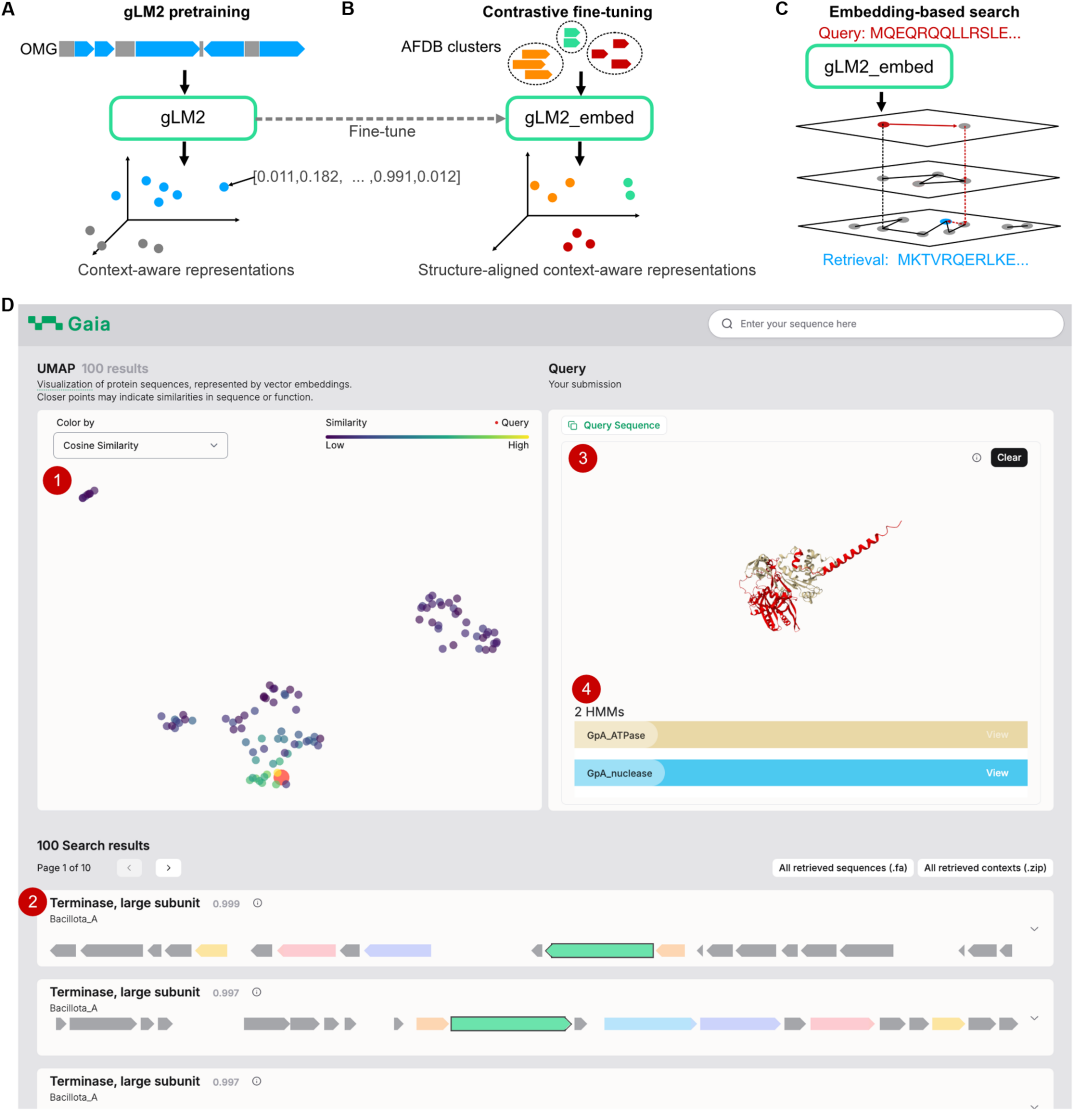
Gaia uses gLM2 representations to perform context-aware search. (**A**) gLM2 is a genomic language model trained on multigene metagenomic contigs (coding sequences shown in blue and noncoding sequences in gray) from the Open MetaGenome (OMG) dataset, with a masked language modeling objective. gLM2 learns context-aware representations of proteins. (**B**) To improve retrieval of remote homologs, we fine-tune gLM2 representations to align them with structural clusters in AlphaFoldDB (AFDB) clusters. (**C**) Gaia’s embedding-based search uses the HNSW algorithm to rapidly find approximate nearest neighbors in a protein vector database embedded by the gLM2_embed model. (**D**) Gaia search web server integrates (1) UMAP (uniform manifold approximation and projection) visualization of retrieved embeddings, (2) visualization of retrieved genomic contexts with top seven most frequently co-occurring genes across the retrieved contigs, (3) ESMFold structure predictions of the query protein as well as all retrieved proteins, and (4) detected Pfam HMMs. Additional information calculated and visualized by Gaia includes local and global alignments and sample metadata (fig. S1).

Gaia enhances protein sequence analysis in genomic datasets by integrating contextual genomic information, a critical dimension often not considered in traditional search methods. The platform’s core is gLM2, a language model trained on the Open MetaGenome (OMG) dataset (*21*). gLM2 is trained on both protein sequences and their surrounding genomic regions, generating features that capture complex relationships between genes and their genomic neighborhoods (i.e., neighboring coding and noncoding sequences, corresponding to 9.7 ± 3.3 genes and represented as 4096 tokens) (Fig. 1A). We fine-tune gLM2 contrastively on 2.3 million structural clusters of the AlphaFoldDB (see Materials and Methods) (*22*), yielding a structure-aligned and context-aware retrieval model (gLM2_embed) (Fig. 1B). gLM2’s context-awareness enables Gaia to identify functionally related proteins that may be missed by sequence-only or structure-only search, such as remote homologs in similar genomic contexts or context-conserved multigene systems (i.e., defense islands and biosynthetic gene clusters).

Gaia search takes in a single protein as a query, which is subsequently embedded into a gLM2_embed vector. We use the hierarchical navigable small-world (HNSW) algorithm to conduct scalable nearest-neighbor search using the cosine similarity metric (Fig. 1C) against a precomputed vector database of 85 million protein clusters (“OG_prot90”, proteins from the OpenGenome dataset clustered at 90% sequence identity). By default, Gaia search returns 100 nearest neighbors from OG_prot90.

Gaia expands the scope of sequence search to include genomic context similarity. To facilitate validation and provide comprehensive insights, Gaia provides a clear visual representation of the genomic architectures of matched sequences with highlighted co-occurring genes and incorporates results from additional tools such as BLASTp identity, Pfam HMM annotations (*23*), and ESMFold for protein structure prediction (Fig. 1D and fig. S1). This integrated approach allows users to efficiently retrieve and analyze relevant sequences, complete with functional annotations, structural predictions, and genomic context visualizations.

**Fig. 2.**
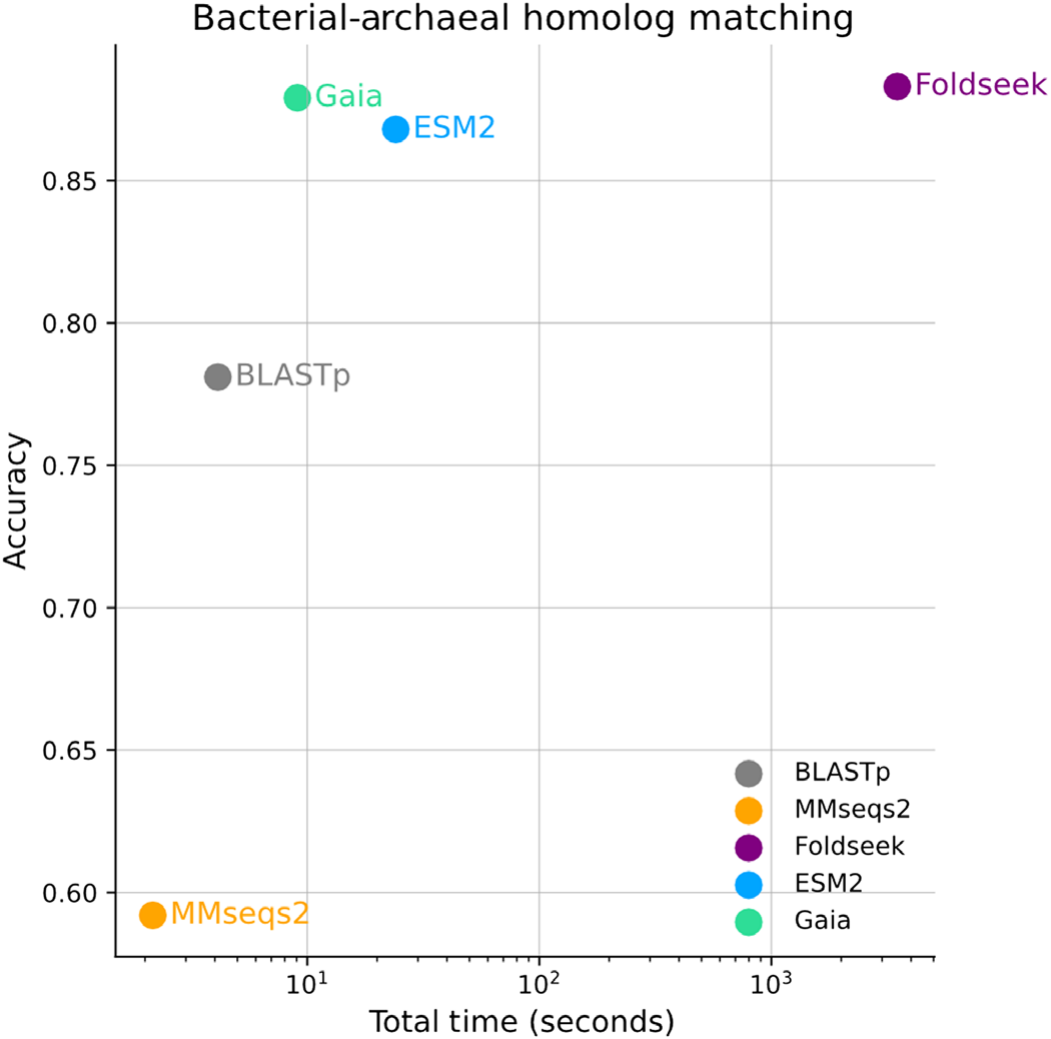
Bacterial-archaeal homolog matching accuracy relative to the time taken for search. The total time includes both search time and database creation time (breakdown shown in table S1) and is shown at the log scale. Accuracy is calculated by counting the correct top one retrieval of the matching archaeal protein given a bacterial protein in a dataset of 256 unique homolog pairs.

We benchmark Gaia search against ESM2 embedding-based search, as well as other commonly used sequence and structural search tools (e.g., BLASTp, MMseqs2, and Foldseek). We compare all five methods for their ability to recover a bacterial-archaeal homolog set (Fig. 2 and table S1), where 256 unique pairs of genes from the *Escherichia coli* K-12 and *Sulfolobus acidocaldarius* DSM 639 genomes were used as a test set to evaluate retrieval accuracy and speed. Gaia demonstrates strong performance in both accuracy and speed for remote homology detection, achieving accuracy comparable to Foldseek while demonstrating greater than two orders of magnitude improvements in speed. Alignment-based methods (BLASTp and MMseqs2), on the other hand, perform poorly in identifying phylogenetically distant homologs. In an additional benchmark using a large and diverse set of proteins, we further break down Gaia’s search sensitivities across three major axes of information pertaining to protein function: sequence (fig. S2A), context (fig. S2B), and structure (fig. S2C). For sequence and context search sensitivity, Gaia search is markedly more sensitive than ESM2-embedding based search (fig. S2A). While embedding-based methods are not as sensitive in retrieving similar sequences and contexts relative to alignment-based methods (BLASTp and MMseqs), they exhibit one to three orders of magnitude improvements in speed (see table S2 for search speed benchmarking against the OG_prot90 database). This makes embedding-based search particularly useful for real-time search. For structural search (figs. S2C and S3), embedding-based methods approach the performance of Foldseek, a structure alignment-based search, without requiring compute-intensive structure prediction. For both structure search and bacteria-archaeal homolog search benchmarks, sequence alignment–based methods are not able to sensitively identify remote homologs. These benchmarks across the three axes indicate that Gaia search is able to retrieve both close and remote homologs, where strong performance in the latter is of particular importance for functional annotations of uncharacterized proteins, but results in a tradeoff with the retrieval of close homologs (fig. S2, A and B). To summarize, Gaia enables rapid and sensitive retrieval of homologs across phylogenetic distances, combining sequence, context, and structural signals. Gaia’s embedding database is dynamic and scalable, addressing search needs for exponentially growing diversity of sequence data (*24*).

**Fig. 3.**
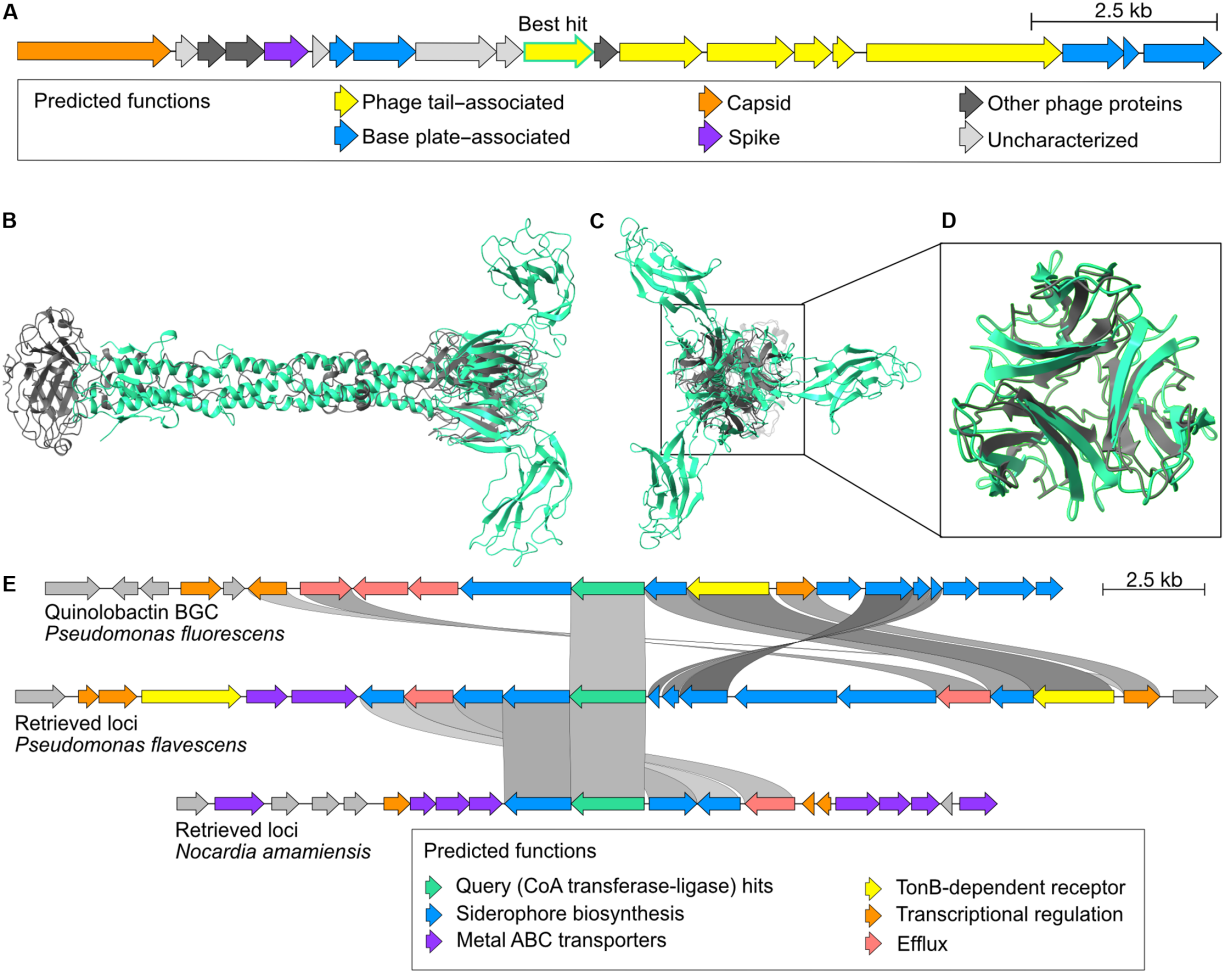
Gaia-enabled discovery of a phage protein. (**A**) Retrieved genomic context of the best hit phage tail protein (green highlight, center) and nearby proteins annotated as bacteriophage proteins by Gaia. (**B**) Superposed trimer structures of the query phage tail protein (green) and a phage pyosin tail fiber protein (PDB: 6CU2; gray) (side view). (**C**) Superposed trimer structures of the query phage tail protein (green) and PDB structure 6CU2 (gray) (front view). (**D**) Aligned portion of the query phage tail protein and reference structure corresponding to the shared C-terminal lectin-like fold. The query is green, and the reference is gray. (**E**) Genome diagrams of the quinolobactin synthesis locus (top), a retrieved sequence from *P. flavescens* (middle), and a retrieved sequence from *N. amamiensis* (bottom). Retrieved sequences were obtained by using the CoA transferase-ligase QbsK (green) as a query.

To demonstrate the utility of Gaia, we provide two case studies of discoveries enabled by Gaia. In our first example, we queried Gaia using a sequence with a previously uncharacterized function (text S1) from *Pseudomonas vancouverensis* LMG 20222. BLASTp matches of this sequence against the National Center for Biotechnology Information “nr” database returns “hypothetical proteins.” Foldseek search of the ESMFold-predicted monomer structure returned proteins of various functions across databases, where lowest *E*-value matches with functional information were “Kinesin-like protein KIN-14F,” “Mediator of RNA polymerase II transcription subunit 21,” “uncharacterized protein,” and “Helix Pomatia agglutinin with no ligands.” Using standard methods of sequence and structural search, the function of this protein is inconclusive. With Gaia search, we first identify that the retrieved sequences occur in genomic contexts, indicative of integrated prophages in bacterial genomes (Fig. 3A and table S3 for genomic context annotations), which is also corroborated by the IMG prediction of the prophage region (*25*). Gaia’s predicted annotation for the best hit of the query protein is “major tail fiber protein S” and was found to likely fold a trimer structure on the basis of pTM (predicted template modeling) and ipTM (interface pTM) scores (ipTM = 0.68; pTM = 0.71) from AlphaFold3. Foldseek search of the trimer structure returned the lowest *E*-value match homology to a trimeric pyosin tail fiber protein from a *Pseudomonas* phage genome [PDB entry 6CU2; query template modeling (qTM) score of 0.17 and template-template modeling (tTM) score of 0.21]. While the direct structural homology (Fig. 3C) between these complexes is limited to a lectin-like domain near the C termini of both proteins (Fig. 3, D and E), both of the fully trimeric structures notably form tail-like structures with highly similar length (Fig. 3C). By comparing 100 retrieved contexts with each other, Gaia finds a diversity-generating retroelement protein Avd (annotated as “Uncharacterized protein UU148”; not present in the best hit context shown in Fig. 3A but frequent in other retrieved contexts) to be among the top seven most frequently co-occurring proteins, further suggesting that this may be a diversity-generating retroelement–associated phage tail protein. Put together, genomic context analysis validates the claim that the query protein is of viral origin and corroborates Gaia’s predicted annotation as a phage tail protein.

In our second example, we chose to search for putative biosynthetic gene cluster (BGC) loci capable of producing siderophores. Such loci are often difficult to differentiate from nonsiderophore BGCs because of the similar chemistry in their synthesis pathways. In our example, we searched for homologous genomic contexts to the quinolobactin biosynthetic gene cluster (MIBiG accession BGC0000925) from *Pseudomonas fluorescens*. Quinolobactin is a small-molecule siderophore with chemical similarity to quinolone compounds, which are used by organisms within the genus *Pseudomonas* for diverse applications including cell-cell signaling (*26*–*28*). The quinolobactin BGC lacks any proteins that contain the IucC condensation domain, the primary signal by which siderophores are identified in antiSMASH (*29*), and so serves as an example of a siderophore locus not easily identifiable with existing BGC identification tools. We used *QbsK* (text S2) CoA transferase-ligase, a key enzyme in the synthesis pathway, from the quinolobactin BGC as a query to Gaia. Gaia-retrieved genomic contexts are putative siderophore loci across phylogenetic distances (Fig. 3E). The first example retrieval from a *Pseudomonas flavescens* genome (same genus as the query) features a distantly homologous (sequence identity of 36 to 55%) set of siderophore synthesis–associated genes in the original quinolobactin locus. The second retrieval example from a much more distant taxon, *Nocardia amamiensis* (different phylum to the query), features a distinctly different siderophore locus from the original query context, which, however, still features siderophore-associated homologous genes with the first retrieval and other characterized siderophore loci from the genus *Pseudomonas* (fig. S4). Such homologous genes associated with siderophore synthesis include *TonB*-dependent transporters with homology to the quinolobactin receptor, salicylate ligase and methylase proteins, and metal-specific ABC transport proteins. The full annotations for selected putative siderophore loci identified by this method are viewable in data S1. These putative synthesis loci identified using Gaia search could not be identified using HMM rule-based methods such as AntiSMASH or with sequence or structural search, as the query protein function (CoA transferase-ligase) is ubiquitous and nonspecific to siderophore synthesis. Through this example, we demonstrate Gaia’s utility for finding previously uncharacterized potential BGCs that share similar genomic architectures.

## DISCUSSION

Gaia provides a unique capability to bioinformatics researchers by incorporating information about the genomic context of proteins. While BLASTp and other sequence alignments approaches excel at finding primary sequence similarities and Foldseek focuses on structural relationships, Gaia provides the ability to identify related proteins with conserved genomic contexts with search speed orders of magnitude faster than existing methods. This capability enables researchers to address complex questions in comparative genomics, such as identifying conserved gene clusters across distantly related organisms, detecting horizontal gene transfer events, and elucidating the function of hypothetical proteins on the basis of their genomic neighborhood. One key limitation of Gaia and other embedding-based search methods is diminished performance for out-of-distribution data (*30*, *31*). For example, Gaia performs worse in retrieval for sequences that were not seen during training (fig. S5), which may be mitigated by fine-tuning on new sequences (*32*). By integrating these approaches, researchers can gain a more comprehensive understanding of protein function and evolution in genomic data, particularly in scenarios where sequence or structural similarity alone may be insufficient.

## MATERIALS AND METHODS

### gLM2 fine-tuning

We fine-tune the pretrained gLM2 model for the retrieval task with a two-stage approach, similar to how text embedding models are fine-tuned from pretrained BERT models (*33*, *34*). In the first stage, we fine-tune gLM2_650M on UniRef50 (https://huggingface.co/datasets/agemagician/uniref50) train split for one epoch. We use the AdamW optimizer, with a learning rate of 1 × 10^−4^ using cosine decay and a batch size of 256. This reduces train-test mismatch as gLM2 is pretrained on genomic contigs instead of individual proteins.

In the second stage, we align representations with the protein structure by training a linear projection layer on mean-pooled, frozen gLM2 representations. We use 2.3 million structural clusters from the AlphaFoldDB (*22*) and train the linear layer to maximize the cosine similarity between proteins in the same cluster. During training, we randomly sample pairs of protein sequences with the same cluster assignment and optimize the InfoNCE contrastive loss (*35*). We use a large batch size of 32,768 to increase the number of in-batch negatives and train for 30,000 steps with a learning rate 1 × 10^−4^ using the AdamW optimizer with a weight decay of 0.1. We use representations from the middle layer of gLM2, as previous studies have shown an improved transfer-learning ability compared to the final layer (*36*, *37*). The output dimensionality of the projection layer is 2.5× smaller than the gLM2 hidden dimension (512 versus 1280 dimensions), improving the scalability of vector search across large databases. gLM2 fine-tuning results in improvements across sequence, context, and structure retrieval sensitivity and bacterial-archaeal homolog matching (table S4).

### OG_prot90 database creation

All protein coding sequences from the OpenGenome dataset (*21*) consisting of 131,744 prokaryotic and viral genomes available on INSDC (International Nucleotide Sequence Database Collaboration) databases (www.insdc.org/) (*38*) and retrieved from IMG/M (*39*) were extracted and clustered at 90% sequence similarity using the MMseqs2 (commit 16e46) (*40*) cluster with configuration --min-seq-id 0.9 -c 0.9, yielding the OG_prot90 database consisting of 85,007,726 centroid sequences. Table S5 compares OG_prot90 database requirements across BLASTp, MMseqs2, ESM2, and Gaia.

### Vector database search

Gaia’s search uses the Qdrant vector database library (*41*) for rapid similarity search against the embedding database. A query protein sequence is first embedded with the gLM2 model, and the resulting vector is used to search for similar sequences in the database using the cosine similarity metric. We use the HNSW algorithm (*42*) to efficiently search for approximate nearest neighbors across millions of protein embeddings. ef_search parameter ablation is found in table S6.

### Gaia output visualization

The output includes a UMAP (uniform manifold approximation and projection) (*43*) representation of the retrieved vector sequences, colored optionally by cosine similarity or by BLASTp percent identity to the input vector. In addition, an ESMfold-generated protein structure (with no recycling) is displayed, along with optional overlays of detected Pfam HMM domains on the query protein. Genome context visualization diagrams, BLASTp identity and coverage, an ESMfold-predicted structure, and a Needleman-Wunsch (*44*, *45*) alignment between the query and each returned subject sequence are available in an expandable accordion for each of the 100 returned subject sequences. HMM annotation in Gaia is performed using pyHMMER (*46*) by running the Pfam HMM database (version 37.0) with the gathering precomputed bitscore thresholds against the query and subject sequences. Predicted protein structures for query and subject sequences are generated using ESMFold and visualized with Molstar (*47*). Coverage values and percent identity were generated with BLASTp using the ncbi-blast+ suite (*1*). Needleman-Wunsch alignment was generated using Biopython (*45*). Top five most frequently co-occurring proteins were calculated by clustering all retrieved context protein embeddings with sklearn (*48*) and DBscan (*49*) with “cosine” metric and eps = 0.01. The Gaia front end was generated using the Nitro UI software package (*50*).

### Functional annotation

Functional annotations for retrieved sequences are generated by aligning pLM representations with functional text annotations. We train a CLIP-like model (*51*) to align ESM2 representations with text annotations, encoded using a pretrained text encoder dmis-lab/biobert-v1.1 (*52*). The model is trained on the Swiss-Prot database for five epochs with a batch size of 6000 protein to text pairs and a learning rate of 1 × 10^−4^. We keep the ESM2 model frozen and train only the projection layer and the text encoder. We precompute functional annotations for each protein in OG_prot90 by finding the closest Swiss-Prot protein embedding in cosine similarity.

### Sequence retrieval sensitivity benchmark

A random set of 1200 sequences was selected from OG_prot90. Sequences were then clustered using CD-HIT 4.8.1 (*53*) with the following parameters: -c 0.5 -n 2. The resulting clustered sequences were then used as a query to BLASTp v2.12.0+ and searched against a BLAST database of OG_prot90. Only 666 sequences with a nonself hit with thresholds of 75% identity and 70% reciprocal coverage were kept. Recall was calculated by determining whether the best nonself BLASTp hit is included in the first *K* retrievals using embedding-based search. For the MMseqs2 baseline, we ran the easy-search pipeline with default parameters.

### Genomic context sensitivity benchmark

A random set of 3000 proteins was selected from OG_prot90 using the seqkit sample function (*54*). We then queried our qdrant database to compare the query protein’s genomic context, where context is defined as up to five protein-coding genes upstream and five protein-coding genes downstream in the genome. We use BLASTp to calculate the homologous fraction of the surrounding proteins between the query and the top 10 retrieved examples, where two proteins are considered homologous if there is sequence identity >50% with coverage >50%. If more than 7 of 10 genes in the retrieved protein’s context are homologous to those in the query protein’s context, we consider the retrieval to be a correct retrieval. For the BLASTp and MMseqs2 baselines, we searched the same 3000 proteins against the OG_prot90 database using default parameters and compared the homologous fractions of the genomic contexts of top 10 nonself hit proteins using the same parameters as above.

### SCOPe-40 retrieval sensitivity benchmark

The SCOPe-40 2.01 (*55*) test set is used to benchmark structural sensitivity, which includes 2207 protein sequences with family, superfamily, and fold structural classification. For each protein sequence, we embed using the gLM2-embed model and retrieve the closest 30 proteins in the test set (excluding self hit) with the cosine similarity metric. We compute recall by checking whether any of the top-*K* retrievals match the query’s structural family. The ESM2 baseline is computed using the middle layer hidden representation from the ESM2 650M model. All other baseline method hits are downloaded from https://wwwuser.gwdguser.de/~compbiol/foldseek/scop.benchmark.result.tar.gz.

### Bacteria-archaeal homolog benchmark

The BacArch BiGene dataset was first curated in (*37*) and is found at https://huggingface.co/datasets/tattabio/bac_arch_bigene. Briefly, *S. acidocaldarius* DSM 639 and *E. coli* K-12 genomes were curated by an expert to identify 256 homologous pairs of protein coding genes with analogous functional annotations. These pairs were manually curated and validated using at least two of five metrics: matching UniRef annotations, genome context similarity, matching HMM annotations, and Foldseek structural similarity. In this study, bacterial proteins were used to search the archaeal protein set, and the top result was used to calculate accuracy. Foldseek was run on ESMfold-generated structures and database creation time (NVIDIA H100 GPU-seconds) were calculated accordingly.

### Out-of-distribution sequence retrieval benchmark

The test split of the UniRef50 dataset (https://huggingface.co/datasets/agemagician/uniref50) was not used for gLM2 pretraining. We removed all noneukaryotic sequences from this set to account for the possibility of similar sequences being included in the pretraining dataset [all metagenomic and likely eukaryotic contigs were removed before dataset creation (*21*)]. The first 1000 sequences were selected to test out-of-distribution effects on sequence retrieval sensitivity, as described above.

### Phage tail protein case study methods

Predicted protein structures for Fig. 2 were generated using AlphaFold3 (*56*). Structure search was performed using the Foldseek-Multimer web service (*57*). The protein reference structure used was obtained from RCSB-PDB (*58*, *59*). The genome context diagram in Fig. 2D was generated using clinker (*60*) from IMG genome ID 2667527228, contig ID Ga0074689_11, part of predicted prophage IMGVR_UViG_2667527228_000002.

### Siderophore locus case study methods

Genome context diagrams were generated using clinker. Reference BGC sequences for loci producing the siderophores quinolobactin (BGC0000925), yersiniabactin (BGC0002570), enterobactin (BGC0000343), and pyochelin (BGC0000412), including their annotations, were obtained from MIBIG (*61*).
