# Supplementary material for: Gaia: An AI-enabled genomic context–aware platform for protein sequence annotation
Source: Sci Adv. 2025 Jun 20;11(25):eadv5109. doi: 10.1126/sciadv.adv5109 (PMC12180486; doi:10.1126/sciadv.adv5109)
Supplement: Supplementary file 1 — Figs. S1 to S5 Tables S1 to S6 Legend for data S1 Text S1 and S2 [file sciadv.adv5109_sm.pdf]

Science Advances
AAAS

Supplementary Materials for

Gaia: An AI-enabled genomic context–aware platform for protein sequence annotation

Nishant Jha *et al.*

Corresponding author: Yunha Hwang, yunha@tatta.bio; Andre Cornman, andre@tatta.bio

Sci. Adv. **11**, eadv5109 (2025)
DOI: 10.1126/sciadv.adv5109

The PDF file includes:

Figs. S1 to S5
Tables S1 to S6
Legend for data S1
Text S1 and S2

Other Supplementary Material for this manuscript includes the following:

Data S1

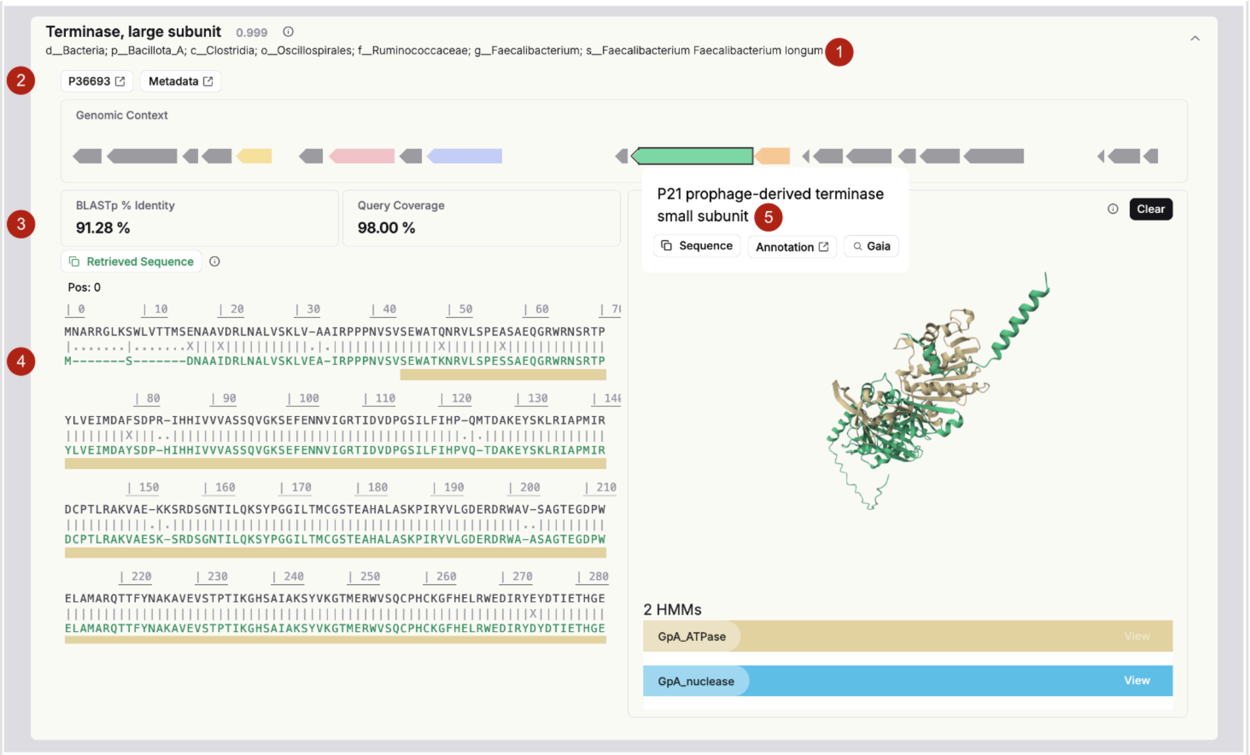

Figure S1. Additional features of Gaia web server. An open accordion view of a search result. (1) Predicted Swiss-Prot annotation with level of confidence (in gray). The full GTDB style taxonomy of the genome this sequence is found is shown below the annotation. (2) Links to the Swiss-Prot annotation this sequence was matched to and metadata information of sample this sequence was found in. (3) BLASTp calculation between the retrieved sequence and the query. (4) Global alignment (Needleman-Wunsch) of the retrieved sequence against the query. (5) Protein-coding genes found in the same context are annotated and can be iteratively searched upon clicking.

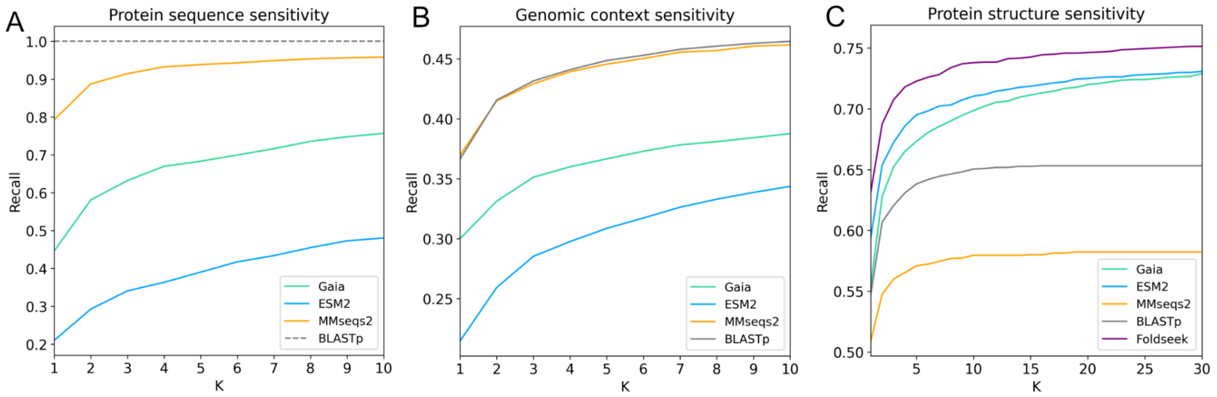

Figure S2. Gaia search sensitivity benchmarks across axes. A) Protein sequence retrieval sensitivity benchmark comparing Gaia and ESM2 embedding based search (n=666), where recall (y-axis) is calculated as the fraction of searches where the best non-self BLASTp match from the OG_prot90 was within the top K retrievals (x-axis). BLASTp is used as the ground truth (recall = 1 across all K), MMseqs2 search (with default settings) is included as a comparison. B) Genomic context retrieval sensitivity benchmark comparing Gaia search and ESM2 embedding-based search (n=3,000), where recall is calculated based on the presence of a gene with a similar genomic context (at least 70% of the proteins in context matching at >50% sequence identity and >50% sequence coverage) within the top K retrievals. We benchmark these methods against MMseqs2 and BLASTp. C) Protein structure-based remote homology retrieval sensitivity benchmark on the SCOPe-40-test dataset (n=2,207). We compare Gaia search and ESM2 embedding-based search sensitivities in retrieving remotely homologous protein families. We benchmark against tools BLASTp, MMseqs2 and Foldseek. We show benchmarking results from other structural class levels in **Fig. S3.**

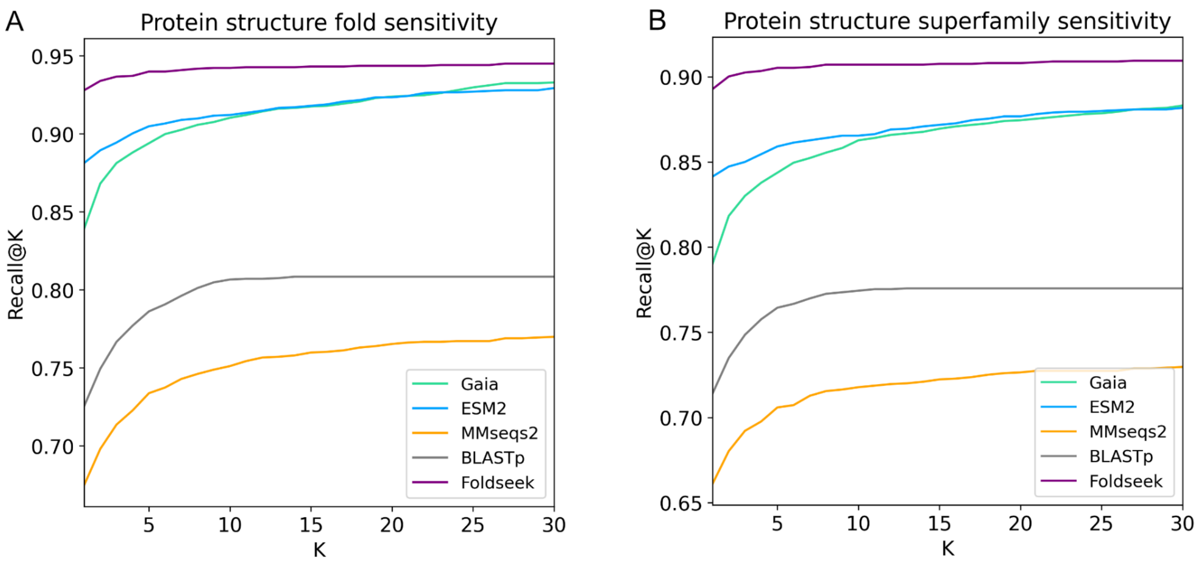

Figure S3. SCOPe-40-test remote homology benchmark at different structural class levels (fold and superfamily).

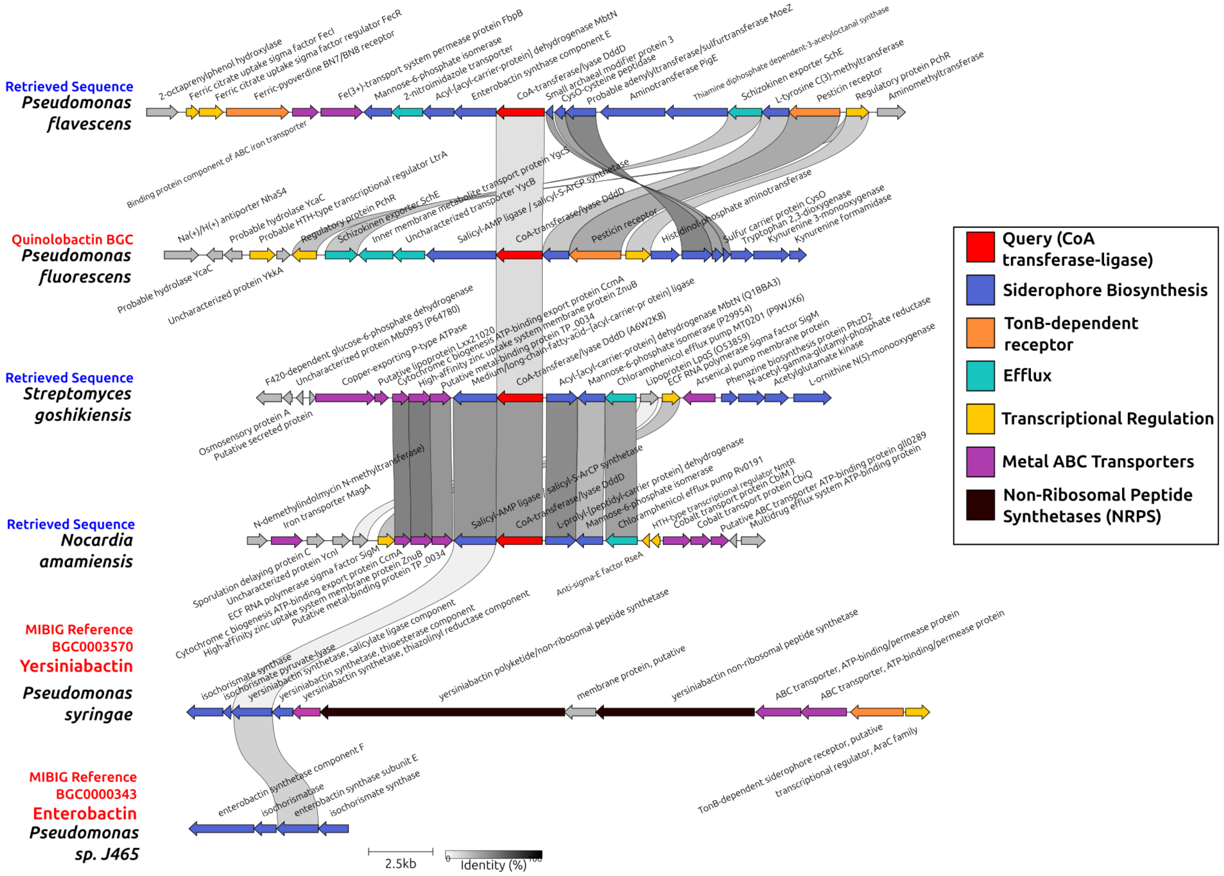

Figure S4. Siderophore genome contexts with reference siderophore loci from the genus *Pseudomonas*. Three retrieved putative siderophore producing loci obtained from searching with Gaia (blue) and three reference siderophore-producing loci from the genus *Pseudomonas* (red). Retrieved sequences were obtained by using Quinolone CoA transferase-ligase QbsK as a query to Gaia. Proteins homologous to the salicylate ligase subunits of Enterobactin and Yersiniabactin synthase were present in all analyzed genome contexts. Putative siderophore producing function in the retrieved sequences is supported by the presence of siderophore receptor-like TonB-dependent receptor genes, metal transport machinery, and efflux pumps with homology to the Schizokinen siderophore efflux system.

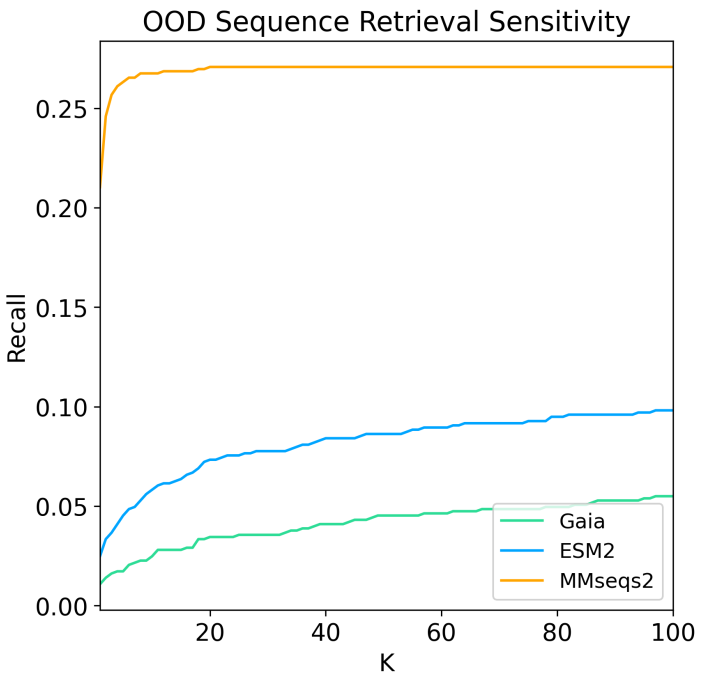

Figure S5. Out of distribution (OOD) sequence retrieval sensitivity. Sequence retrieval performance (using BLASTp as ground truth as in Fig S2A) for 1000 Eukaryotic protein sequences that were not included in pretraining nor finetuning of gLM2. These proteins were included in ESM2 training, resulting in improved retrieval performance.

Table S1. Bacteria-Archaea remote homology benchmark. We compare the search methods on the accuracy of correctly retrieving functional homologs in an archaeal genome (*Sulfolobus acidocaldarius* DSM 639 ASM1228v1) given a gene in a bacterial genome (*Escherichia coli* K-12). ESMfold was used to predict structures for Foldseek. We report retrieval accuracy, the time taken to generate the database (GPU-seconds on 1 NVIDIA H100 GPU) and the search time.

	BLASTp	MMseqs2	Foldseek	ESM2	Gaia
Accuracy	0.781	0.592	0.883	0.868	0.879
GPU-seconds	-	-	3,480	24	9
Search time	4.12	2.16	8.6	0.07	0.07

Table S2. Speed benchmarking. We compare embedding-based search methods against BLASTp and MMseqs2 for searching 1, 10, 100, 1,000 sequences against OG_prot90. Target database (OG_prot90) creation times are not included in this calculation. BLASTp was run with the default settings using 20 threads. MMseqs2 was run with default settings with index in memory (--db-load-mode 2 after touchdb) and 20 threads. Gaia includes GPU inference time for sequence embedding (T4 GPU) and vector search using Qdrant with default HNSW parameters (m=16, ef_construct=100) and 20 threads, with k=100 nearest neighbors. Time is reported in seconds.

No. of sequences	BLASTp	MMseqs2	Gaia
**1**	18.7	8.3	**0.14**
**10**	98.2	15.3	**0.2**
**100**	1070	21.3	**1.3**
**1000**	10768	32.5	**14**

Table S3. Full predicted annotations for the genomic context of putative phage tail protein. Annotations for the genomic context of the putative phage tail protein highlighted in **Figure 3**.

Protein_ID	Annotation	PFAM_Hits
2667641139	Major capsid protein	CLP_protease (score=123.1, e=1.3e-38); Mu-like_gpT (score=31.1, e=1.7e-10)
2667641140	SPbeta prophage-derived uncharacterized protein YonC	No PFAM hits
2667641141	Head-tail joining protein	No PFAM hits
2667641142	Mu-like prophage FluMu protein gp37	No PFAM hits
2667641143	Spike protein	Phage_base_V (score=56.7, e=2.7e-18)
2667641144	Uncharacterized 7.7 kDa protein in gp5-gp4 intergenic region	No PFAM hits
2667641145	Baseplate protein W	GPW_gp25 (score=65.7, e=2.8e-21)
2667641146	Baseplate protein J	Baseplate_J (score=79.1, e=3.5e-25)
2667641147	Uncharacterized protein YqcA	Tail_P2_I (score=107.6, e=5.5e-34)
2667641148	Uncharacterized protein YqcB	No PFAM hits
2667641149	Major tail fiber protein S	No PFAM hits
2667641150	Collar protein p132	No PFAM hits
2667641151	Tail fiber receptor-binding protein	No PFAM hits
2667641152	Putative prophage major tail sheath protein	Phage_sheath_1 (score=100.2, e=1.2e-31); Phage_sheath_1C (score=82.5, e=2.1e-26)
2667641153	Tail tube protein	Phage_tube (score=195.7, e=5.1e-61)
2667641154	Tail assembly protein E	Phage_TAC_7 (score=54.2, e=1.3e-17)
2667641156	Probable tape measure protein	PhageMin_Tail (score=78.0, e=9.7e-25)
2667641157	Baseplate tail-tube junction protein gp48	Phage_P2_GpU (score=121.1, e=2.2e-38)
2667641158	Baseplate protein X	Phage_tail_X (score=72.3, e=2.1e-23)
2667641159	Probable baseplate hub protein	Phage_GPD (score=68.0, e=8.9e-22)
2667641160	Glycosyl hydrolase family 19 domain-containing protein HI_1415	Glyco_hydro_19 (score=38.7, e=1.1e-12)

Table S4. Retrieval sensitivity benchmarking before and after finetuning gLM2.

	Bac-Arch Homolog Accuracy	Sequence recall (k=10)	Context recall (k=10)	Structure recall (k=30)
gLM2 (before finetune)	0.82	0.65	0.33	0.63
gLM2_embed (after finetune)	0.88	0.76	0.39	0.73

Table S5. HNSW ef_search parameter ablation on the sequence retrieval benchmark.

ef_search	32	64	128	256	512	1024	2048
Sequence recall (k=10)	0.725	0.737	0.751	0.764	0.764	0.766	0.766
Search Speed per query	0.06	0.08	0.11	0.27	0.47	0.90	1.52

Table S6. OG_prot90 database requirements for search methods. Note that Foldseek is not included as it was not feasible to create a predicted structure database for OG_prot90.

	BLASTp	MMseqs2	ESM2	Gaia
Index creation time (minutes)	13	16	30	22
Peak RAM (GB)	25	200	45	35
Disk (GB)	36	243	219	98

Data S1. full annotations for selected putative siderophore loci identified Pfam annotations, Gaia annotations for retrieved sequences, and MIBIG annotations for reference sequences for the genomic contexts of retrieved putative siderophore producing loci and reference sequences from MiBIG are provided Gaia_Additional_Data_1.xlsx.

Text S1. Putative Phage Protein Sequence

```
MANLNGATGVAAIFKAFLRKLETTDPKHPDTWNPNYQTLIDNDVFLKAFADEVSTARGSQPSLKDRLVAI
EQTQASLSPEYIDELTAAVKYALDQAGVANRSIRALKSQLQQEGELLIENRGIVSGCTATKSTTAARNLN
LAAGVCFANGRAYSVDSGNNMASVPSNISAGNASAVVYLYRSGNGWKMAVTAIGEAVPAGAIRLYNVTIP
PNSTDATDPTLANVTLTSVRRVEVGFPQYLDTPVSQFVAINNLSANDFRLDFEVVSAEGAPCERKSLSVP
SRATNGFTLELASAADNVLVRYRVSKLNN
```

Text S2. QbsK protein sequence used as query for siderophore loci discovery

```
>Pseudomonas_fluorescens_QbsK

MSLLKHLTLHLSASAWSAELAPVAEALARRLKAQGGQVATGGQTLCDDAGHQVQLHLRPWPAPSTVAASA
ALVEAVVGLTALHQRSSGEPLPLGVDYCATFTASLLLTAALASLLGQARGLPAARLAMSHGGAALLAIGQ
YLAMDSADGGYPPEPAPPADAVRPPFTSADGVVFELEALNPDAWLRFWQQAGVDIAVAGKGWRPFMQRYT
RATAWLPAALMRAASEHDFAQLQAMAKEAGTALCALQRWQDCRNQPVFQPWIQSGGWRCTEFAPGPGVNG
PDEQQPPQHAGLPLQGITVVECCRLIQGPLAGHVLRLLGATVIKVEPPGGDPMRGMPPMAGEISAHFDAI
NRSKQTVQIDLKAPTGRAELLALLAHADVFLHNWAPGRDEQLALQPHTLARLLPRLIYVSASGTGPAPTS
DQPLGTDFMIQAYSGLAERIRQPHGAAGGALITLVDVLGAVAAAEGIVAALYARQRDGRGRYLDSCMAGA
AALLLAGQPDGQAPEPAEAFACRDGWLMLDRAQSARGRDGLARLQADPGLAPWCAEQDTHACCRALQAMG
VMAVAVTADLRQLPDLPLLAGALRQSAYLHVNNPWEISNL
```
